# Sex- and region-biased depletion of microglia/macrophages attenuates CLN1 disease in mice

**DOI:** 10.1186/s12974-020-01996-x

**Published:** 2020-10-28

**Authors:** Kristina Berve, Brian L. West, Rudolf Martini, Janos Groh

**Affiliations:** 1 Department of Neurology, Section of Developmental Neurobiology, University Hospital Würzburg, Würzburg, Germany; 2 Present address: Theodor-Kocher-Institute, University of Bern, Bern, Switzerland; 3 Plexxikon Inc., Berkeley, CA USA

**Keywords:** Neuronal ceroid lipofuscinosis, Microglia, Macrophages, T lymphocytes, Neurodegeneration, Axon degeneration

## Abstract

**Background:**

The neuronal ceroid lipofuscinoses (CLN diseases) are fatal lysosomal storage diseases causing neurodegeneration in the CNS. We have previously shown that neuroinflammation comprising innate and adaptive immune reactions drives axonal damage and neuron loss in the CNS of palmitoyl protein thioesterase 1-deficient (*Ppt1*^−/−^) mice, a model of the infantile form of the diseases (CLN1). Therefore, we here explore whether pharmacological targeting of innate immune cells modifies disease outcome in CLN1 mice.

**Methods:**

We applied treatment with PLX3397 (150 ppm in the chow), a potent inhibitor of the colony stimulating factor-1 receptor (CSF-1R) to target innate immune cells in CLN1 mice. Experimental long-term treatment was non-invasively monitored by longitudinal optical coherence tomography and rotarod analysis, as well as analysis of visual acuity, myoclonic jerks, and survival. Treatment effects regarding neuroinflammation, neural damage, and neurodegeneration were subsequently analyzed by histology and immunohistochemistry.

**Results:**

We show that PLX3397 treatment attenuates neuroinflammation in CLN1 mice by depleting pro-inflammatory microglia/macrophages. This leads to a reduction of T lymphocyte recruitment, an amelioration of axon damage and neuron loss in the retinotectal system, as well as reduced thinning of the inner retina and total brain atrophy. Accordingly, long-term treatment with the inhibitor also ameliorates clinical outcomes in CLN1 mice, such as impaired motor coordination, visual acuity, and myoclonic jerks. However, we detected a sex- and region-biased efficacy of CSF-1R inhibition, with male microglia/macrophages showing higher responsiveness toward depletion, especially in the gray matter of the CNS. This results in a better treatment outcome in male *Ppt1*^−/−^ mice regarding some histopathological and clinical readouts and reflects heterogeneity of innate immune reactions in the diseased CNS.

**Conclusions:**

Our results demonstrate a detrimental impact of innate immune reactions in the CNS of CLN1 mice. These findings provide insights into CLN pathogenesis and may guide in the design of immunomodulatory treatment strategies.

**Supplementary information:**

**Supplementary information** accompanies this paper at 10.1186/s12974-020-01996-x.

## Background

Neuronal ceroid lipofuscinosis (CLN) diseases are a group of rare inherited lysosomal storage diseases characterized by substantial neural damage in the CNS, culminating in neuron loss, mental and physical disability, and eventually leading to an early death [6, 41]. Different subtypes of the CLNs are caused by mutations in distinct genes implicated in endo-lysosomal and cellular homeostasis [8, 9, 56]. The CLNs are histopathologically characterized by the accumulation of lipofuscin-like autofluorescent storage material (ceroid) in most cell types of the CNS, neuroinflammation and neurodegeneration [56]. Despite recent progress regarding possible therapeutic avenues, current disease management is still mostly restricted to mitigating or controlling disease symptoms [36, 41]. Many of the CLN subtypes manifest in childhood with an early onset and a fast disease progression. Visual impairment caused by degeneration of the retinotectal system is usually one of the earliest symptoms and steadily develops into blindness, resulting in an isolated and desperate situation for the affected individuals and families. Mutations in the palmitoyl-protein thioesterase 1 (PPT1), a soluble lysosomal enzyme, result in the devastating infantile form called CLN1 [34].

We could previously show in PPT1-deficient (*Ppt1*^−/−^*)* mice [30] that secondary neuroinflammation, comprising innate and adaptive immune reactions in the CNS, acts as potent disease modifier amplifying axonal perturbation, neurodegeneration, and clinical outcome [25, 27]. In these studies, proof-of-principle cross-breeding and bone marrow transplantation experiments unequivocally demonstrated that CD8^+^ T lymphocytes drive axon degeneration in the CNS and disease progression [25]. Targeting these adaptive immune reactions by pharmacological treatment approaches using the immune modulators fingolimod or teriflunomide, both clinically established drugs for the treatment of multiple sclerosis, attenuated neuroinflammation and improved disease outcome [22]. Moreover, we could show in the same mouse model that pro-inflammatory, activated microglia/macrophages interact with CD8^+^ T lymphocytes and thereby promote disease progression [25, 27]. The latter study identified a central role of the myeloid cell surface molecule sialoadhesin (Sn; CD169; Siglec-1) in this interaction, thereby promoting axonal perturbation, neuron loss, as well as clinical outcome in the CLN1 disease model [27]. Increased expression of Sn by microglia/macrophages negatively controlled the number of CD8^+^CD122^+^ regulatory T lymphocytes in the CNS which inhibit adaptive immune reactions by limiting CD8^+^CD122^−^ cytotoxic effector T cell reactions [1, 27, 48]. These observations link innate and adaptive immune reactions [19, 27, 57, 73] in which microglia might be one of the primary promoters of neuroinflammation by increased Sn-mediated interaction with T cells in CLN1. Other studies also implicated dysfunctional pro-inflammatory myeloid reactions with defects in CLN-related genes [43, 50, 58].

Considering the described detrimental role of microglia/macrophage reactions observed in CLN disease models, targeting these cells using a pharmacological attempt might not only offer insights into their pathological role within the diseased CNS but furthermore provides a valuable tool for prospective therapeutic strategies aiming to mitigate disease burden. We recently applied a CSF-1R inhibitor targeting CSF-1R-dependent microglia in another hereditary CNS disease model. Mice carrying point mutations in the *PLP1* gene that have previously been found in multiple sclerosis patients [20, 23, 72] were successfully treated with PLX3397 which attenuated neuroinflammation-related neural damage [24], adding to the growing evidence that microglial reactions play important roles in a plethora of CNS disorders comprising genetically mediated diseases [26, 65].

In the present study, we treated *Ppt1*^−/−^ mice with the same CSF-1R inhibitor resulting in a significant reduction of neuroinflammatory reactions as well as an attenuation of neurodegeneration and an improvement of clinical features. However, we encountered a striking sex- and CNS compartment-related bias in treatment efficacy in *Ppt1*^−/−^ mice. These observations emphasize the contextual and regional heterogeneity of microglia/macrophages [32, 52, 69] and furthermore highlight the need for a more detailed analysis of sex- and brain region-specific differences with regards to assessments of prospective treatment approaches.

## Methods

### Animals

Mice were kept in the animal facility of the Department of Neurology under barrier conditions and at a constant cycle of 12 h in the light (< 300 lx) and 12 h in the dark. All animal experiments were approved by the Government of Lower Franconia, Germany. *Ppt1*-deficient (*Ppt1*^−/−^) mice carried a disruption of exon 9 [30]. *Ppt1*^−/−^ mice as well as age-matched wildtype (*Wt*) littermates were on a uniform C57BL/6J genetic background. Genotypes were determined by conventional PCR using isolated DNA from ear punch biopsies following previously published protocols [30].

### PLX3397 treatment and tissue preservation

PLX3397 (provided by Plexxikon Inc., Berkeley, CA, USA) was prepared as a 150 ppm drug chow to dose ~ 27 mg PLX3397/kg body weight when given ad libitum. This was based on our previous short-term dose finding and long-term treatment approaches in which we observed efficient microglia depletion without obvious neurological side effects in *Wt* mice [24]. Control mice received normal chow without the pharmacological inhibitor. Mice were treated for 5 months with daily monitoring concerning certain burden criteria and phenotypic abnormalities. The preventive treatment approach started at 1 month of age before the first symptoms of the disease occur. No deleterious side effects but an increase in body weight and a variable change in fur colour were detected upon treatment. After the treatment mice were euthanized with CO2 (according to guidelines by the State Office of Health and Social Affairs Berlin) and transcardially perfused with phosphate-buffered saline (PBS) containing heparin followed by 2% paraformaldehyde (PFA) in PBS for 10 min. Tissue was collected, post-fixed in 2% PFA for 1 h, dehydrated using 30% sucrose in PBS overnight, embedded in Tissue-Tek OCT compound (Sakura), and frozen in isopentane cooled by liquid nitrogen. Before dehydration of the brains, olfactory bulbs and medullae were separated at defined positions, and total brains, including pontes, were weighed using an analytical balance (ABT 220-5DM, Kern).

### Histochemistry and Immunofluorescence

Immunohistochemistry was performed on 10-μm-thick longitudinal optic nerve cryo-sections after post-fixation in 4% PFA in PBS or ice-cold acetone for 10 min. Sections were blocked using 5% bovine serum albumin (BSA) in PBS and incubated overnight at 4 °C with one or an appropriate combination of up to three of the following antibodies: rat anti-CD4 (1:1000, Bio-Rad AbD Serotec), rat anti-CD8 (1:500, Bio-Rad AbD Serotec), rat anti-CD11b (1:100, Bio-Rad AbD Serotec), rat anti-CD169 (1:300, Bio-Rad AbD Serotec), mouse anti-SMI32 (1:1000, BioLegend), rabbit anti-Tmem119 (1:500, Abcam), rabbit anti-APP (1:1000, Abcam), rabbit anti-CSF-1R (1:100, SantaCruz). Immune reactions were visualized using fluorescently labeled (1:300, Dianova) secondary antibodies, streptavidin (1:300, Invitrogen), or biotinylated secondary antibodies (1:100, Vector Laboratories) and streptavidin–biotin–peroxidase (Vector Laboratories) complex using diaminobenzidine-HCl and H_2_O_2_), and nuclei were stained with DAPI (Sigma-Aldrich). Moreover, 40-μm-thick coronal brain sections were used for free-floating immunohistochemistry using the same antibodies. Coronal brain sections were treated with 0.3% Sudan Black B for 5 min after secondary antibody incubation to quench autofluorescent signals. Light and fluorescence microscopic images were acquired using an Axiophot 2 microscope (Zeiss) with an attached CCD camera (SPOT Imaging; Diagnostic Instruments, Inc.) or an Axio imager.M2 microscope (Zeiss) with ApoTome.2 structured illumination equipment, attached Axiocam cameras, and corresponding software (ZEN 2.3 blue edition). Images were minimally processed (rotation, cropping, addition of symbols) for generation of figures using Photoshop CS6 (Adobe). For quantification, immunoreactive profiles were manually counted in at least three nonadjacent sections for each animal and related to the area of these sections using the cell counter plugin in Fiji/ImageJ (National Institutes of Health). For quantification of retinal ganglion cells, specific markers were labeled in free-floating retina preparations. Fixed retinae were frozen in PBS containing 2% Triton X-100, thawed, washed, and blocked for 1 h using 5% BSA and 5% donkey serum in PBS containing 2% Triton X-100. Retinae were incubated overnight on a rocker at 4 °C with guinea pig anti-RBPMS antibody (1:300, Merck), a specific marker of RGCs [62]. Immune reactions were visualized using fluorescently labeled (1:500, Dianova) secondary antibodies, retinae were flat mounted, RGC densities were manually quantified, and the total retinal area was measured.

### Semiquantitative real-time PCR

After transcardial perfusion with PBS containing heparin, optic nerves were quickly dissected, snap frozen in liquid nitrogen, and stored at − 80 °C until further processing. Nerves were homogenized (ART-MICCRA D-8, ART Labortechnik) in TRIzol reagent (Invitrogen) and total RNA was isolated according to the manufacturers’ guidelines. Concentration and quality of RNA were determined using a BioPhotometer (Eppendorf) and 1 μg of RNA was reverse transcribed in a 100 μl reaction using random hexamer primers (Applied Biosystems). cDNA samples were analyzed as duplicates by semiquantitative real-time PCR using predeveloped TaqMan assays (IL-1β: Mm00434228_m1; TNFα: Mm00443258_m1; CXCL10 Mm00445235_m1; GAPDH as internal standard, Mm99999915_g1) and TaqMan universal PCR master mix (Applied Biosystems) according to the manufacturer’s guidelines.

### Flow cytometry

Mice were transcardially perfused with PBS containing heparin, brains were dissected, collected in ice-cold PBS, and cut into small pieces. Tissue was digested in 1 ml Accutase (Merck) per brain at 37 °C for 30 min and triturated through 100 μm cell strainers which were rinsed with 10% FCS in PBS. Cells were purified by a linear 40% Percoll (GE Healthcare) centrifugation step at 650 g without brakes for 25 min and the myelin top layer and supernatant were discarded. Mononuclear cells were resuspended in FACS buffer (1% BSA and 0.1% sodium azide in PBS) and counted. Viable cells were identified by Live/Dead stain (Thermo Fisher), Fc receptors were blocked for 15 min with rat anti-CD16/32 (1:100, BD Pharmingen), and cells were washed and labeled with the following antibodies for 30 min at 4 °C: rat anti-CD11b APC (1:100, BioLegend), rat anti-CD45 FITC (1:100, BioLegend), rat anti-Siglec H PE (1:100, BioLegend). Cells were washed twice, single viable cells were gated, and analyzed using a FACSCalibur (BD Biosciences) and FlowJo (version 10) software.

### Spectral domain optical coherence tomography

Mice were subjected to optical coherence tomography (OCT) imaging with a commercially available device (Spectralis OCT; Heidelberg Engineering) and additional lenses as previously described [28]. Mice were measured at different ages for longitudinal analysis and the thickness of the innermost retinal composite layer comprising nerve fiber layer (NFL), GCL, and inner plexiform layer (IPL) were measured in high-resolution peripapillary circle scans (at least 10 measurements per scan) by an investigator unaware of the age group and genotype of the mice.

### Analysis of visual acuity

Mice were analyzed regarding visual acuity using automated optokinetic reflex tracking in an OptoDrum device with the corresponding software (Striatech), as previously described [3]. Briefly, mice were placed on an elevated platform surrounded by monitors and a stripe pattern with maximum contrast and constant rotation speed (12°/s) was presented. Behavior was automatically detected and analyzed by the software in an unbiased manner and the stimulus pattern (cycles) was continuously adjusted to find the threshold of the animal’s visual acuity.

### Accelerating rotarod analysis

Mice were placed on a RotaRod Advanced system (TSE systems), and the time on the constantly accelerating rod (5 to 50 rpm; max latency 300 s) was measured in five consecutive runs per trial as previously described [23]. Mice were trained with two trials on two consecutive days and measured in a third trial on the third day.

### Analysis of myoclonic jerks and longevity

The frequency of myoclonic jerks was observed during the daylight cycle by placing mice individually in a clean cage in a quiet room for a 5-min observation period. During this period, an observer unaware of genotype documented all myoclonic jerks according to described criteria [30]. Kaplan-Meier analysis was used to measure cumulative survival and determine differences in lifespan. Moribund mice were sacrificed according to guidelines by the State Office of Health and Social Affairs Berlin.

### Experimental design and statistical analysis

All quantifications and behavioral analyses were performed by investigators unaware of the genotypes of the respective mice after concealment of genotypes with individual uniquely coded labels. Animals were randomly placed into experimental or control groups according to genotyping results using a random generator (http://www.randomizer.org). For biometrical sample size estimation, the program G*Power (version 3.1.3) was used [17]. Calculation of appropriate sample size groups was performed in a priori power analysis by comparing the mean of two groups with a defined adequate power of 0.8 (1—beta-error) and an α-error of 0.05. To determine the pre-specified effect size *d*, previously published data were considered as comparable reference values [25]. Statistical analysis was performed using GraphPad Prism (version 7) software. Shapiro-Wilk test was used to check for normal distribution of data. Normally distributed data were compared using the two-tailed unpaired Student’s *t* test. For multiple comparisons, one-way ANOVA followed by Tukey’s post hoc tests (parametric comparison) or Kruskal-Wallis tests with Bonferroni correction (non-parametric comparison) were applied. *P* values considered as significant were indicated by asterisks or hashtags according to the following scheme: *^, #^*P* < 0.05; **^, ##^*P* < 0.01; ***^, ###^*P* < 0.001. Significant differences of a respective group in comparison with untreated wildtype mice are indicated above the corresponding bar. Exceptions are the quantifications separating and comparing female and male sex. Here, hashtags above the bars indicate significant differences to untreated *Ppt1*^−/−^ mice of the same sex. Each symbol (♂: male, ♀: female, ○: male or female) represents the mean value of one mouse. Some datasets are shown separately for sex because there were obvious differences between female and male samples detected.

## Results

### Microglia/macrophage depletion using PLX3397 reveals sex- and CNS region-specific bias in *Ppt1*^−/−^ mice

**Fig. 1:**
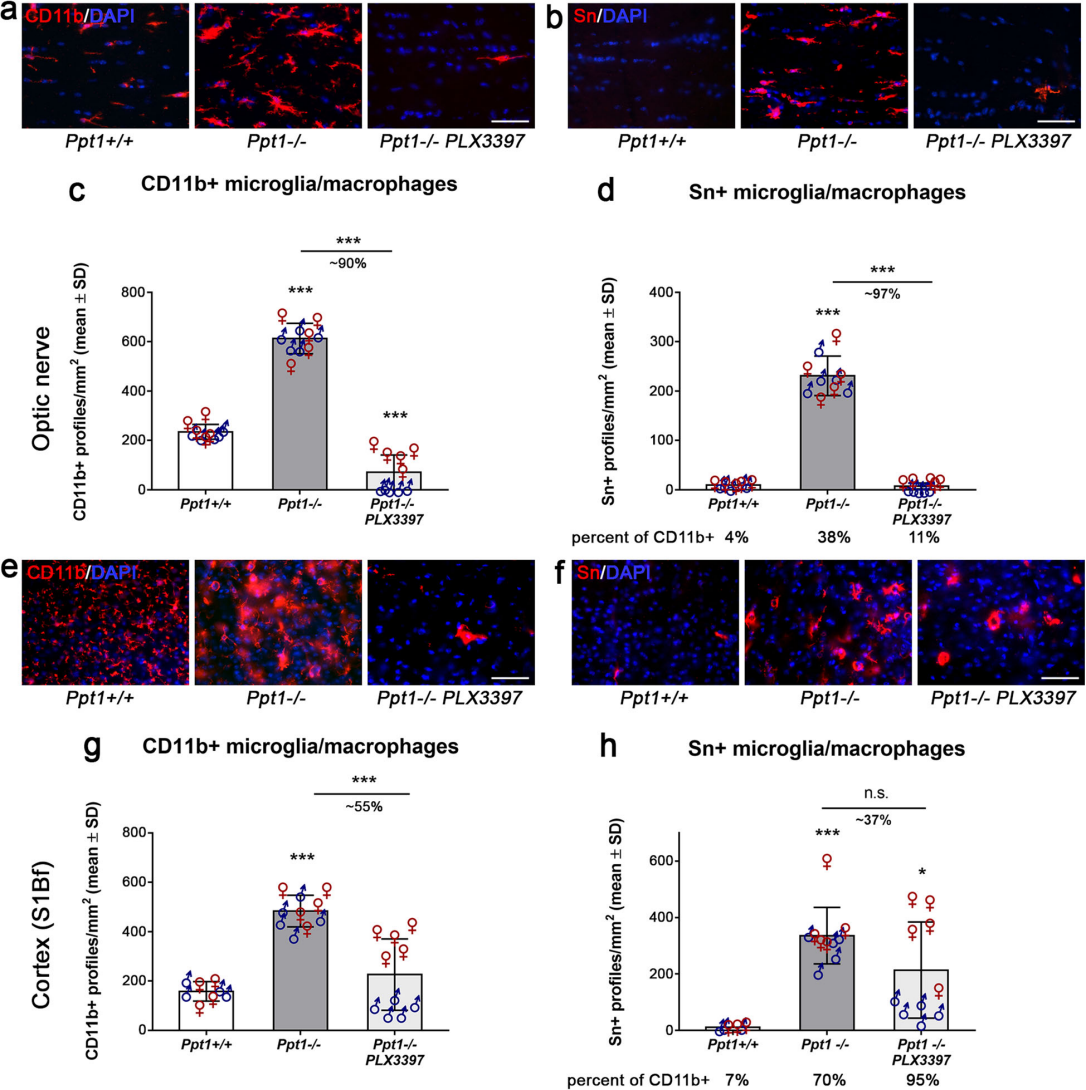
Region-biased depletion of CD11b+ and Sn+ cells in the CNS of CLN1 mice upon treatment with PLX3397. **a**, **b** Representative fluorescent microscopic images of immunohistochemically labeled CD11b^+^ or activated Sn^+^ microglia/macrophages in longitudinal optic nerve sections of 6-month-old *Ppt1*^+/+^, *Ppt1*^−/−^, and PLX3397-treated *Ppt1*^−/−^ mice after 5 months of treatment. Scale bar: 50 μm. **c**, **d** Quantification of CD11b^+^ or Sn^+^ cells showed a significant reduction in number in optic nerves after PLX3397 treatment. **e**, **f** Representative fluorescent microscopic images of immunohistochemically labeled CD11b^+^ or activated Sn^+^ microglia/macrophages in S1Bf cortex region of 6-month-old *Ppt1*^+/+^, *Ppt1*^−/−^ mice, and PLX3397-treated *Ppt1*^−/−^ mice after 5 months of treatment. Scale bar: 50 μm. **g**, **h** Quantification of CD11b^+^ or Sn^+^ cells showed a significant or non-significant tendential reduction in number in the S1Bf cortex region after PLX3397 treatment, respectively. Percentages of Sn^+^ cells related to CD11b^+^ cells are indicated below the corresponding groups. *n* = 5 male and 5 female mice per group. One-way ANOVA and Tukey’s post hoc tests. *P < 0.05; ***P < 0.001

We recently showed that targeting microglia using long-term oral administration of the CSF-1R inhibitor PLX3397 depletes microglia without obvious neurological side effects and significantly attenuates neuroinflammation-related neural damage in the CNS of a genetic model displaying features characteristic of progressive multiple sclerosis and hereditary spastic paraplegia [23, 24]. We here used the same approach to deplete microglia/macrophages in the CNS of *Ppt1*^−/−^ mice. After long-term treatment for 5 months, we quantified the number of CD11b^+^ microglia/macrophages in optic nerve and somatosensory barrel field (S1Bf) cortex sections of *Ppt1*^+/+^ (wildtype), *Ppt1*^−/−^ mice, and PLX3397-treated *Ppt1*^−/−^ mice (Fig. 1a, c, e, g). As previously shown [22, 25, 27], CD11b is a reliable pan marker of CNS myeloid cells and CD11b^+^ cells were elevated in number in untreated *Ppt1*^−/−^ compared to *Ppt1*^+/+^ mice (Fig. 1c, g). After treatment with PLX3397, the numbers of CD11b^+^ cells were significantly reduced in both CNS compartments, the retinotectal system and S1Bf cortical region of *Ppt1*^−/−^ mice. Consequently, in longitudinal optic nerve sections, microglia/macrophages expressing the pro-inflammatory myeloid cell surface molecule sialoadhesin (Sn) were significantly reduced in number after PLX3397 treatment (Fig. 1b, d). However, Sn^+^ microglia/macrophages in cortical regions only showed a minor non-significant decrease after treatment (Fig. 1f, h). The depletion of activated microglia/macrophages in optic nerves was reflected by a prominent reduction in the mRNA expression of the pro-inflammatory cytokines IL-1β, TNFα, and CXCL10 (Supplementary Figure 1).

Interestingly, during these quantifications, we encountered a clear bias in depletion efficacy of mutant mice regarding different brain regions and sex. While there was a robust depletion of CD11b^+^ cells in the optic nerve by approximately 90%, the reduction of CD11b^+^ cells in the cortical region was limited to approximately 55%. CD11b^+^ cells in the distinct CNS compartments of *Ppt1*^−/−^ mice also showed profound differences in cell morphology. While in optic nerves of *Ppt1*^−/−^ mice, CD11b^+^ cells showed thicker “bushy” processes as previously described [25]; there were hyper-ramified “bushy” cells and less ramified amoeboid cells detectable in cortical regions (Supplementary Figure 2), likely indicating stronger phagocytic activity [66]. Especially CD11b^+^ amoeboid cells appeared resistant to PLX3397 treatment in the cortex of *Ppt1*^−/−^ mice. Since a considerable amount of the remaining microglia/macrophages in the cortex region of *Ppt1*^−/−^ mice expressed the pro-inflammatory activation marker Sn (Fig. 1h), these findings reveal a strongly activated treatment-resistant myeloid population, especially in this compartment.

**Fig. 2:**
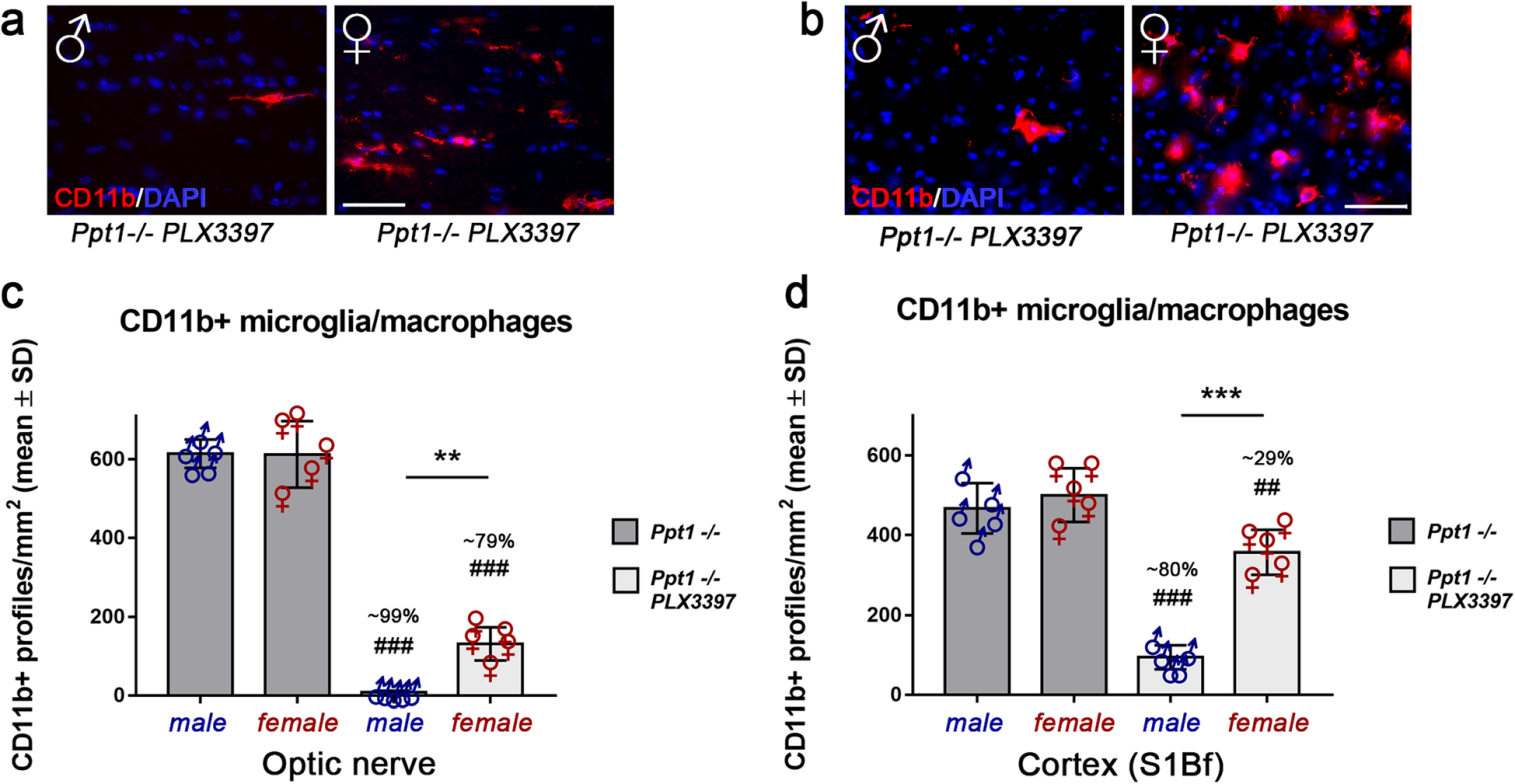
Sex-biased depletion of CD11b+ cells in the CNS of CLN1 mice upon treatment with PLX3397. **a**, **b** Representative fluorescent microscopic images of immunohistochemically labeled CD11b^+^ microglia/macrophages of male and female PLX3397-treated *Ppt1*^−/−^ mice in longitudinal optic nerve and S1Bf cortex sections, respectively. Scale bar: 50 μm. **c**, **d** Quantification of CD11b^+^ cells showed a significant sex bias, with a higher percentage of depletion in male *Ppt1*^−/−^ mice treated with PLX3397. *n* = 5 male and 5 female mice per group. One-way ANOVA and Tukey’s post hoc tests. **^, ##^*P* < 0.01; ***^, ###^*P* < 0.001. # significant difference to untreated *Ppt1*^−/−^ mice of the same sex. Datasets also shown in Fig. 1 are presented separately for sex

Additionally, a sex bias favoring the depletion of microglia/macrophages in male *Ppt1*^−/−^ mice was detected in both CNS compartments (Fig. 2a, b, c, d). This sex bias resulted in a significant difference in the number of remaining microglia/macrophages comparing male and female mutants after PLX3397 treatment and again was especially pronounced in the cortex region. Untreated *Ppt1*^−/−^ mice showed no obvious sex differences regarding microglia/macrophage numbers or morphology (Fig. 2a–d).

### High levels of CSF-1R and TMEM119 expression on CD11b^+^ cells correlate with more efficient depletion in the cortex region of *Ppt1*^−/−^ mice

**Fig. 3:**
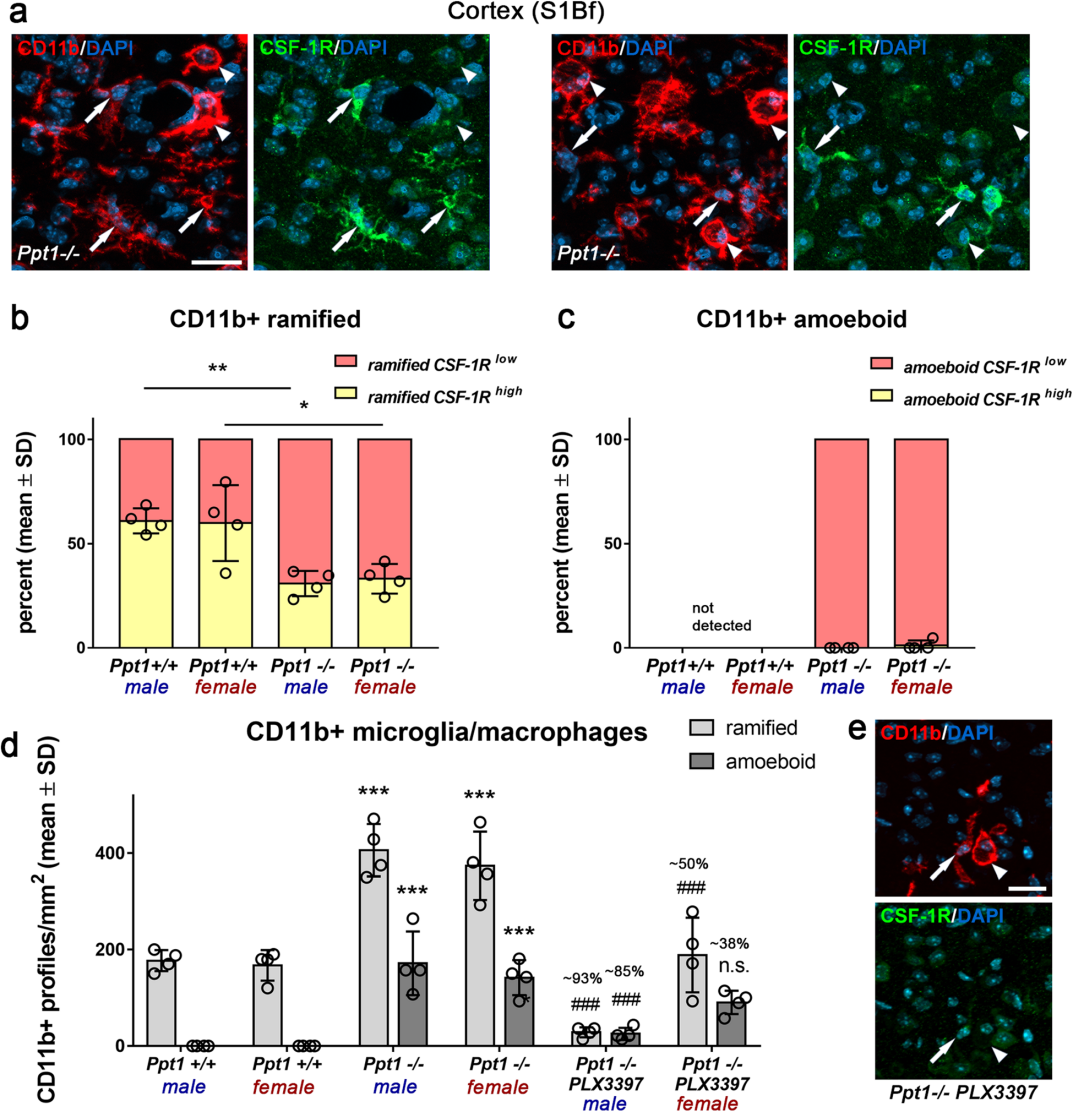
High levels of CSF-1R expression correlate with efficient depletion of CD11b^+^ cells in the cortex of CLN1 mice. **a** Representative fluorescent microscopic images of immunohistochemical labeling of CSF-1R on CD11b^+^ microglia/macrophages in the S1Bf cortex of untreated 6-month-old *Ppt1*^−/−^ mice. Arrows: ramified (and hyper-ramified) cells; arrowheads: amoeboid cells. Scale bar: 20 μm. **b**, **c** Quantification of CSF-1R immunoreactivity on ramified and amoeboid CD11b^+^ microglia/macrophages in *Ppt1*^*+/+*^ and *Ppt1*^*+/+*^ mice showed that high CSF-1R expression was restricted to ramified CD11b^+^ cells. In addition, the percent of ramified cells highly expressing the CSF-1R was significantly reduced in untreated *Ppt1*^−/−^ compared with *Ppt1*^*+/+*^ mice. Two-tailed unpaired Student’s *t* test. *P < 0.05; **P < 0.01. **d** Quantification of ramified and amoeboid CD11b^+^ cells in *Ppt1*^+/+^, *Ppt1*^−/−^, and PLX3397-treated *Ppt1*^−/−^ mice showed that in mutant mice ramified cells were depleted more efficiently compared with amoeboid cells. *n* = 4 male and 4 female mice per group. One-way ANOVA and Tukey’s post hoc tests. ***^, ###^*P* < 0.001. # significant difference to untreated *Ppt1*^−/−^ mice of the same sex. **e** Representative fluorescent microscopic images of immunohistochemical labeling of CSF-1R on CD11b^+^ microglia/macrophages in the S1Bf cortex of a 6-month-old PLX3397-treated *Ppt1*^−/−^ mouse. Arrow: ramified cell; arrowhead: amoeboid cell. Scale bar: 20 μm

Next, we focused on the cortex region and challenged the hypothesis that the encountered sex bias in depletion efficacy might be associated with differential expression levels of CSF-1R on male versus female microglia/macrophages of *Ppt1*^−/−^ mice. We categorized the activation state of cortical microglia by morphological criteria and determined the corresponding CSF-1R intensity by immunohistochemistry (Fig. 3a). We found that the amount of CD11b^+^ cells in the cortex region of untreated *Ppt1*^−/−^ as well as *Ppt1*^+/+^ mice expressing high levels of CSF-1R was not dependent on sex (Fig. 3b, c). Instead, in both sexes, activation state partially determined CSF-1R intensity, with only a certain number of ramified cells expressing higher levels CSF-1R. Of note, this amount was lower in *Ppt1*^−/−^ mice in comparison to *Ppt1*^+/+^ mice, indicating that microglial activation leads to downregulation of CSF-1R. Consequently, amoeboid microglia/macrophages, as indicative for strong activation state and phagocytic load, were confined to *Ppt1*^−/−^ mice and exclusively expressed CSF-1R to a low extend (Fig. 3a, c). Finally, quantification of ramified and amoeboid CD11b^+^ microglia/macrophages after PLX3397 treatment could show that in mutant mice ramified microglia/macrophages were depleted more efficiently resulting in a shift toward remaining amoeboid microglia/macrophages with low CSF-1R expression in the treated group (Fig. 3d, e). Overall, these results suggest that the activation state and degree of CSF-1R expression found on microglia/macrophages correlate with an efficient depletion but do not explain the observed sex bias.

We also considered the possibility that monocyte-derived myeloid cells infiltrate the cortex region of *Ppt1*^−/−^ mice. Therefore, we used an antibody against the microglia-specific marker TMEM119 [5] to determine the identity of ramified and amoeboid CD11b^+^ cells. Interestingly, few of the ramified and almost all amoeboid-shaped CD11b^+^ cells in the cortex region of *Ppt1*^−/−^ mice were TMEM119 negative while virtually all ramified cells seen in wildtype mice were positive for this marker (Supplementary Figure 3). This indicates that CD11b^+^ amoeboid cells in the cortex of *Ppt1*^−/−^ mice are comprised of either highly activated microglia that downregulate TMEM119 (see below) and/or infiltrated monocyte-derived macrophages. Flow cytometry of freshly isolated CD11b^+^ cells from whole brains supported the latter hypothesis as there was an increased frequency of non-microglial myeloid cells (CD11b^+^ CD45high SiglecH-) detectable in *Ppt1*^−/−^ compared with *Ppt1*^*+/+*^ mice (Supplementary Figure 4).

### Microglia/macrophage depletion using PLX3397 significantly reduces recruitment of T cells in the CNS of *Ppt1*^−/−^ mice

**Fig. 4:**
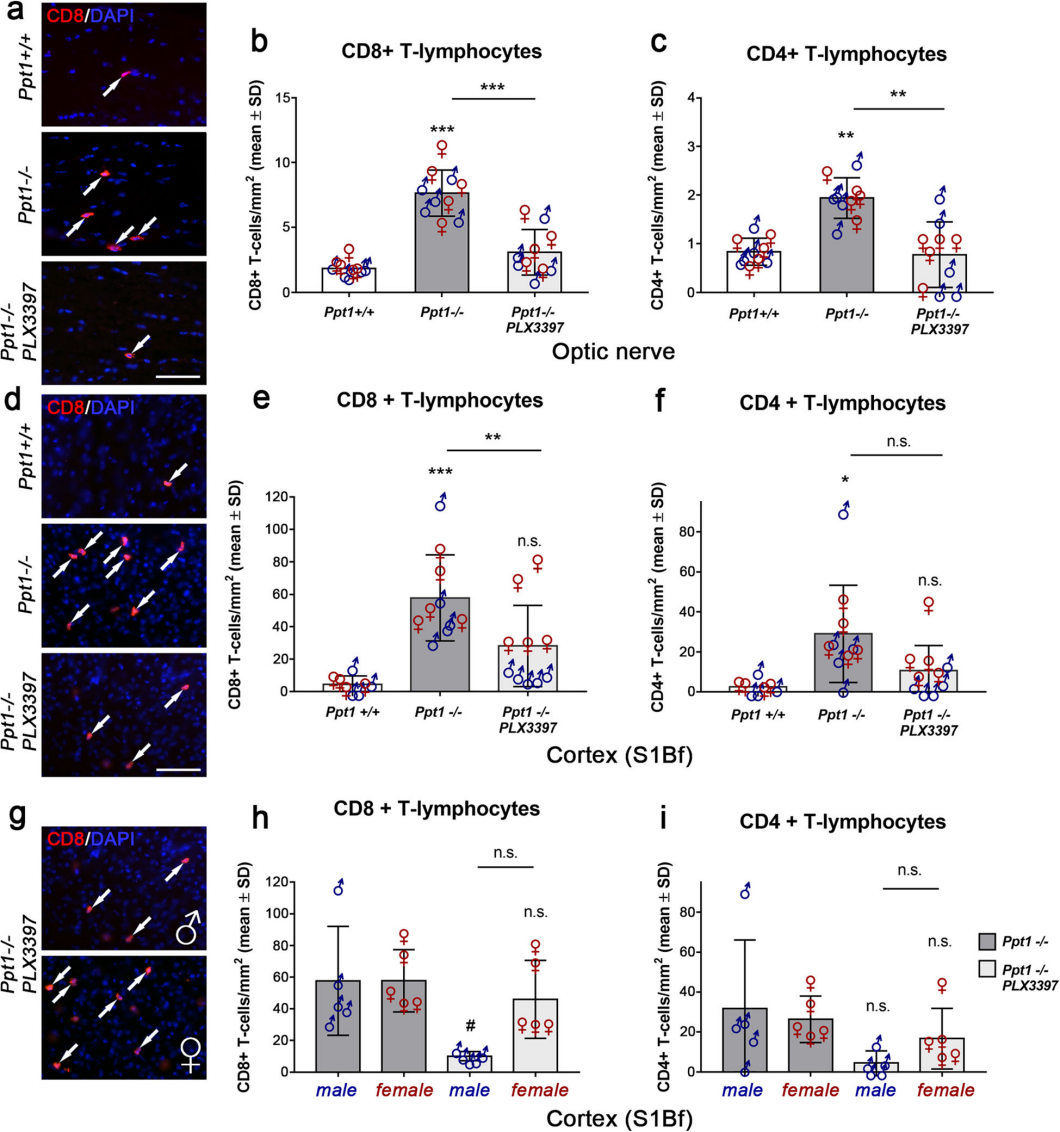
Depletion of microglia/macrophages attenuates the recruitment of T lymphocytes in the CNS of CLN1 mice. **a** Representative fluorescent microscopic images of immunohistochemically labeled CD8^+^ T cells (arrows) in longitudinal optic nerve sections of 6-month-old *Ppt1*^+/+^, *Ppt1*^−/−^, and PLX3397-treated *Ppt1*^−/−^ mice after 5 months of treatment. Scale bar: 50 μm. **b**, **c** Quantification of CD8^+^ or CD4^+^ T cells in optic nerves of 6-month-old *Ppt1*^+/+^, *Ppt1*^−/−^, and PLX3397-treated *Ppt1*^−/−^ mice showed a significant reduction of T cell numbers after microglia/macrophage depletion. **d** Representative fluorescent microscopic images of immunohistochemically labeled CD8^+^ T cells in the S1Bf cortex region. Scale bar: 50 μm. **e**, **f** Quantification of CD8^+^ or CD4^+^ T cells in the S1Bf cortex showed a significant reduction of CD8^+^ T cell numbers in PLX3397-treated *Ppt1*^−/−^ mice. **g** Representative fluorescent microscopic images of immunohistochemically labeled CD8^+^ T cells in S1Bf cortex of male and female PLX3397-treated *Ppt1*^−/−^ mice. **h**, **i** Quantifications of CD8^+^ or CD4^+^ T cells in the S1Bf cortex region of *Ppt1*^−/−^ and PLX3397-treated *Ppt1*^−/−^ mice separated by their sex revealed a strong sex bias favoring the reduction of CD8^+^ T cell numbers in male treated mice. *n* = 5 male and 5 female mice per group. One-way ANOVA and Tukey’s post hoc tests. *^, #^*P* < 0.05; **P < 0.01; ***P < 0.001. # significant difference to untreated *Ppt1*^−/−^ mice of the same sex

As previously observed in a different disease model [24], treatment with PLX3397 also indirectly attenuated the numbers of CD8^+^ and CD4^+^ T lymphocytes in the CNS of *Ppt1*^−/−^ mice (Fig. 4a–f) which have previously been shown to be increased [22, 25]. The reduction was significant for CD8^+^ T lymphocytes (Fig. 4a, b, d, e), a cell population of the adaptive immune system shown to be the predominant subtype infiltrating the CNS of *Ppt1*^−/−^ mice and driving axonal damage [25]. Consistent with a role of microglia reactions in T cell recruitment and the differential efficacy of microglia/macrophage depletion in male versus female mice, numbers of CD8^+^ and CD4^+^ T cells were differentially attenuated as well in the cortex region (Fig. 4c, f, i). In male treated mice, the number of remaining T cells was lower compared to the female counterparts, an effect that was more pronounced for CD8^+^ T cells and not detectable in the optic nerve of the same mice (Fig. 4a, b).

### Microglia/macrophage depletion using PLX3397 attenuates axonal damage and neuron loss in the CNS of *Ppt1*^−/−^ mice

**Fig. 5:**
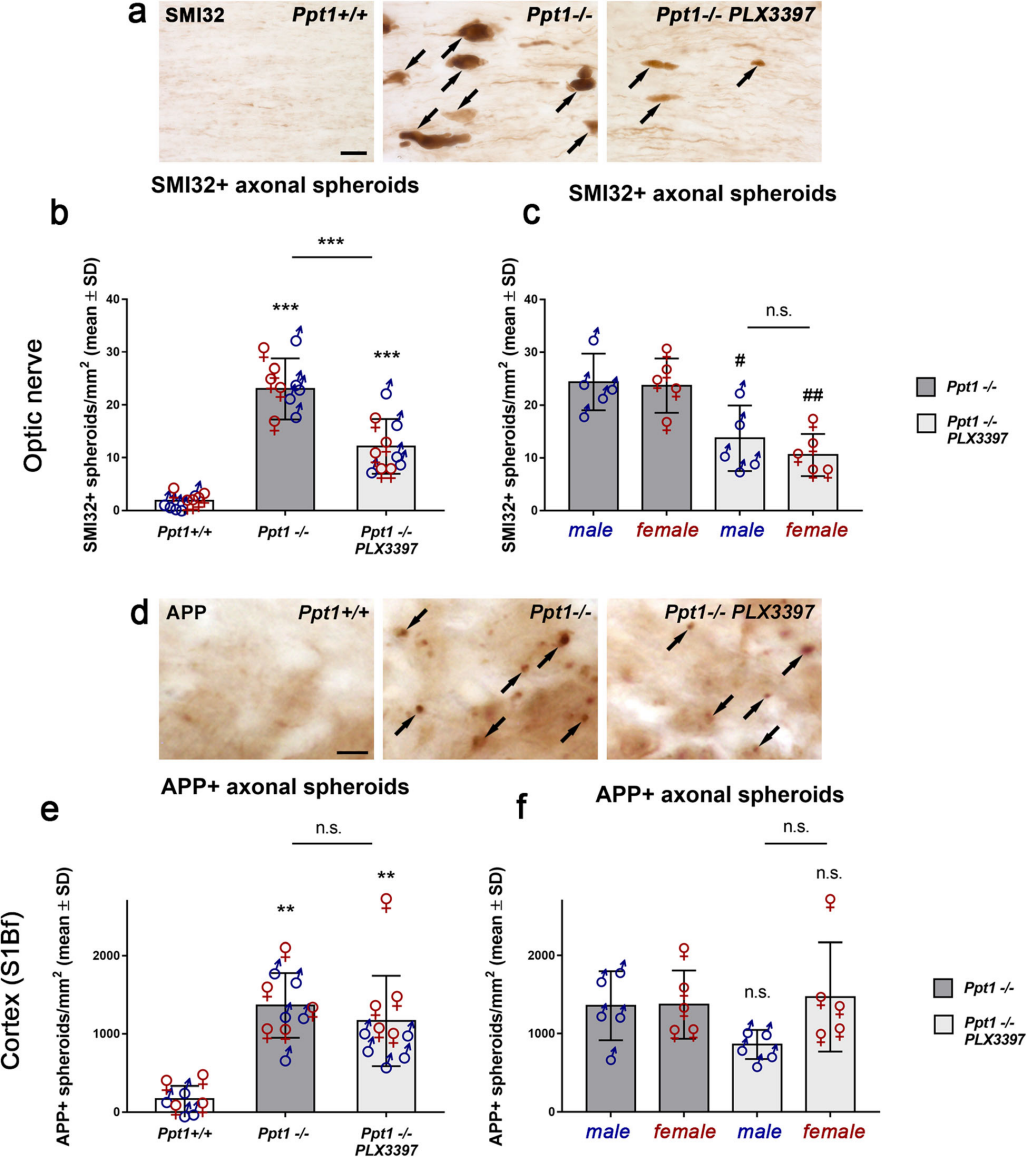
Depletion of microglia/macrophages attenuates axon damage in the CNS of CLN1 mice. **a** Immunohistochemical detection of SMI32+ axonal spheroids (arrows) in longitudinal optic nerve sections of 6-month-old *Ppt1*^+/+^, *Ppt1*^−/−^ mice and PLX3397-treated *Ppt1*^−/−^ mice. Scale bar: 20 μm. **b** Quantification of SMI32+ axonal spheroids optic nerves showed a significant reduction of ongoing axonal damage after microglia depletion. **c** Quantification of SMI32+ axonal spheroids of *Ppt1*^−/−^ and PLX3397-treated *Ppt1*^−/−^ mice separated by their sex did not show any differences regarding axonal spheroids. **d** Immunohistochemical detection of APP+ axonal spheroids (arrows) in the S1Bf cortex region of 6-month-old *Ppt1*^+/+^, *Ppt1*^−/−^, and PLX3397-treated *Ppt1*^−/−^ mice. Scale bar: 20 μm. **e** Quantification of APP+ axonal spheroids in the S1Bf cortex region revealed that microglia/macrophage depletion did not significantly attenuate ongoing axonal damage within this CNS compartment. **f** Quantification of APP+ axonal spheroids in the cortex separated by sex showed a non-significant tendency to reduction of spheroid numbers in male but not female PLX3397-treated *Ppt1*^−/−^ mice. *n* = 5 male and 5 female mice per group. One-way ANOVA and Tukey’s post hoc tests. ^#^*P* < 0.05; **^, ##^*P* < 0.01; ***P < 0.001. # significant difference to untreated *Ppt1*^−/−^ mice of the same sex

Regarding histopathological changes, we first quantified SMI32^+^ or amyloid precursor protein (APP^+^) axonal spheroids indicative of ongoing axonal damage in longitudinal sections of optic nerves or coronal sections of the lower S1Bf cortex region (Fig. 5). As previously described, prominent axonal spheroid formation was detected in *Ppt1*^−/−^ mice [22, 25, 27]. PLX3397 treatment significantly reduced the number of SMI32^+^ profiles in optic nerves of male and female *Ppt1*^−/−^ mice (Fig. 5a–c). In contrast to this, pharmacological depletion of microglia/macrophages could not significantly attenuate the number of APP^+^ axonal spheroids in the cortex region (Fig. 5d, e). This was most likely due to the less efficient microglia/macrophage depletion and corresponding mild attenuation of neuroinflammation in the cortical regions especially of female mice (Fig. 5f).

**Fig. 6:**
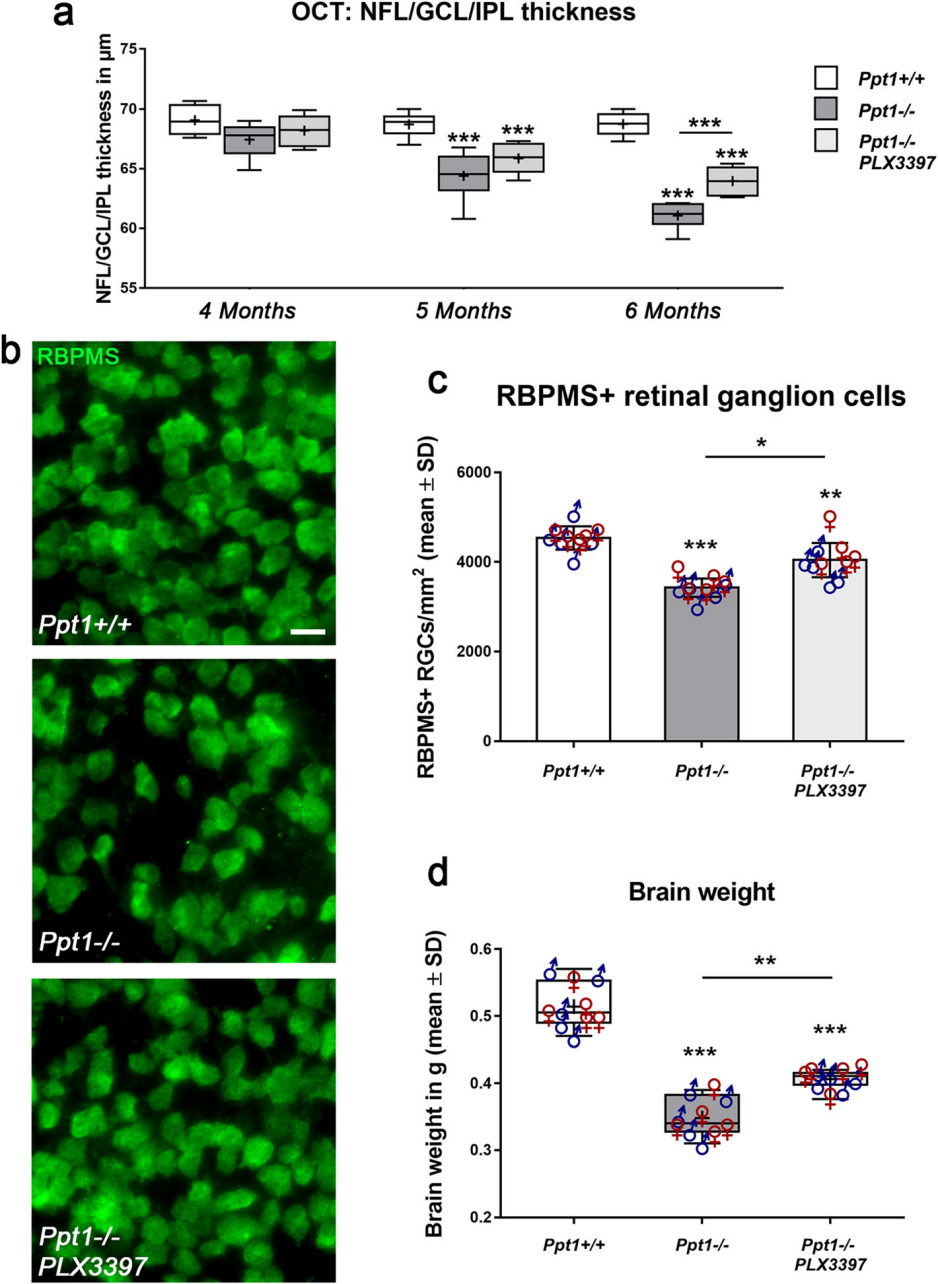
Depletion of microglia/macrophages reduces retinal thinning and ganglion cell loss and attenuates brain atrophy in CLN1 mice. **a** Longitudinal OCT analysis of the innermost retinal composite layer thickness in *Ppt1*^+/+^, *Ppt1*^−/−^, and PLX3397-treated *Ppt1*^−/−^ mice from 4 to 6 months of age. Retinal thinning was significantly delayed and less pronounced in PLX3397-treated *Ppt1*^−/−^ mice. **b** Representative fluorescent microscopic images of immunohistochemically labeled RBPMS+ retinal ganglion cells in flat mounts of 6-month-old *Ppt1*^+/+^, *Ppt1*^−/−^ mice and PLX3397-treated *Ppt1*^−/−^ mice. Scale bar: 20 μm. **c** Quantification of RBPMS+ retinal ganglion cells showed a significant reduction of neuron loss in PLX3397-treated *Ppt1*^−/−^ mice. **d** Analysis of total brain weight showed that depletion of microglia/macrophages resulted in attenuation of brain atrophy in PLX3397-treated CLN1 mice. *n* = 5 male and 5 female mice per group. One-way ANOVA and Tukey’s post hoc tests. *P < 0.05; **P < 0.01; ***P < 0.001

In line with a significant reduction of axonal damage in the retinotectal system of *Ppt1*^−/−^ mice, PLX3397 treatment reduced thinning of the retina as measured by longitudinal OCT analysis of living mice (Fig. 6a). Retinal thinning showed a non-significant tendency toward attenuation in PLX3397 treated *Ppt1*^−/−^ mice at 4 and 5 months of age which reached significance at 6 months. Moreover, a beneficial effect on the survival of retinal ganglion cells was detected (Fig. 6b, c). Similarly, and irrespective of sex, we found a significant attenuation of brain atrophy as reflected by decreased reduction in total brain weights of *Ppt1*^−/−^ mice treated with PLX3397 (Fig. 6d).

### Microglia/macrophage depletion using PLX3397 leads to significant amelioration of the clinical phenotype and increased longevity of *Ppt1*^−/−^ mice

**Fig. 7:**
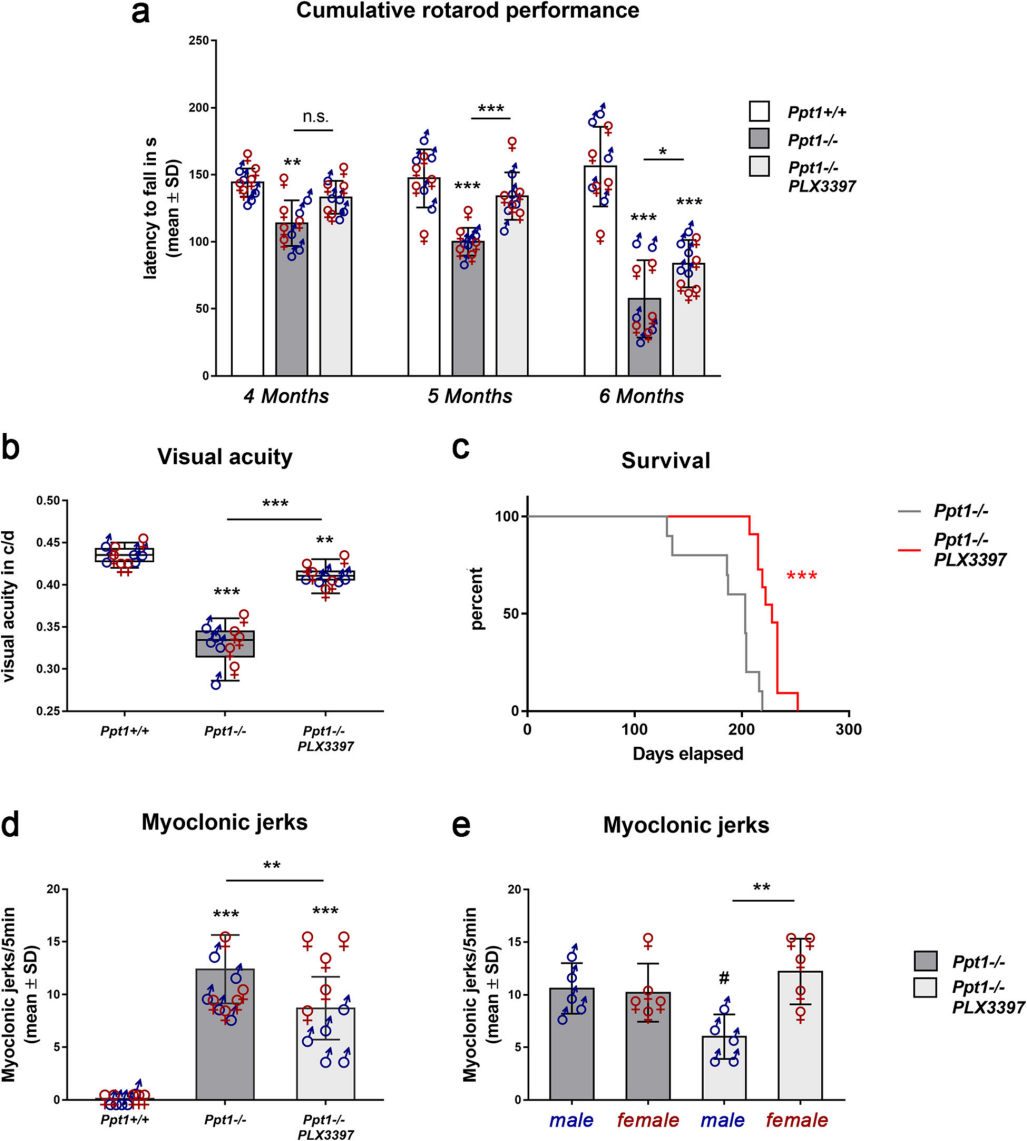
Depletion of microglia/macrophages improves clinical outcome in CLN1 mice. **a** Longitudinal analysis of cumulative rotarod performance from 4 to 6 months of age in *Ppt1*^*+/+*^, *Ppt1*^−/−^, and PLX3397 treated *Ppt1*^−/−^ mice. Motor impairment was attenuated in PLX3397 treated *Ppt1*^−/−^ mice. **b** Visual acuity measured by optokinetic responses showed that PLX3397-treatment significantly improved visual acuity in *Ppt1*^−/−^ mice. **c** Kaplan-Meier survival analysis demonstrated increased longevity of CLN1 mice upon treatment. Log-rank test. **d**, **e** Quantification of myoclonic jerks in 6-month-old *Ppt1*^*+/+*^, *Ppt1*^−/−^, and PLX3397-treated *Ppt1*^−/−^ mice showed that their frequency in *Ppt1*^−/−^ mice was significantly reduced upon PLX3397 treatment. This treatment effect was more pronounced in male mutants compared to their female counterparts. *n* = 5 male and 5 female mice per group. One-way ANOVA and Tukey’s post hoc tests. *^, #^*P* < 0.05; **P < 0.01; ***P < 0.001. # significant difference to untreated *Ppt1*^−/−^ mice of the same sex

To investigate the functional outcome of microglia/macrophage depletion in *Ppt1*^−/−^ mice, we longitudinally analyzed motor coordination by accelerating rotarod. There was a progressive and significant decrease in *Ppt1*^−/−^ mice compared with wildtype mice detectable and this decline was significantly attenuated upon long-term PLX3397 treatment (Fig. 7a). Moreover, we analyzed visual acuity using automated unbiased optokinetic tracking. As previously reported [25], we found that the visual acuity was significantly decreased in 6-month-old *Ppt1*^−/−^ mice and this decrease was attenuated in *Ppt1*^−/−^ mice treated with PLX3397 (Fig. 7b). Also, the lifespan of *Ppt1*^−/−^ mice treated with PLX3397 was mildly but significantly increased compared with untreated controls (Fig. 7c)

Interestingly, we again detected a treatment-related sex bias regarding myoclonic jerks, a typical clinical outcome measure of *Ppt*^−/−^ mice [30]: While the frequency of jerks in untreated males and females was similar, male mice responded to treatment with a significant reduction of jerks, while females did not (Fig. 7d). This observation is in line with the less efficient attenuation of neuroinflammation in specific CNS compartments of female mice (Figs. 2d, 3d, and 4h, i).

## Discussion

This study provides further evidence for a detrimental contribution of the innate immune system within disease course and outcome in a mouse model of a lysosomal storage disorder. We could show that the pharmacological depletion of microglia/macrophages in the CNS using the CSF-1R inhibitor PLX3397 significantly attenuates the neuroinflammatory response and thereby ameliorates neurodegeneration and clinical outcome in *Ppt1*^−/−^ mice, a model of CLN1.

In detail, long-term inhibition of CSF-1R signaling resulted in an overall attenuation of microglial activation, reduction of pro-inflammatory cytokine expression, as well as a decreased recruitment of T lymphocytes, mostly of the CD8^+^ subtype, in the CNS of CLN1 mice. Correspondingly, this led to a significant reduction of axonal damage, neuron loss, brain atrophy, and an amelioration of clinical disease.

### Efficacy of microglia/macrophage depletion depends on sex and CNS compartment

Surprisingly, we encountered a robust bias regarding depletion efficacy among sex and different CNS compartments (retinotectal system vs. cortex region) during our investigations. Microglia/macrophages in female *Ppt1*^−/−^ mice were less responsive to administration of PLX3397 resulting in a less pronounced improvement of the overall neuropathological phenotype. This was particularly obvious in the cortex, where female *Ppt1*^−/−^ mice showed no attenuation of microgliosis, CD8^+^ T cell recruitment, and axon damage upon the treatment. Concerning the clinical readouts, myoclonic jerks were significantly more frequent in female treated mice compared to their male counterparts while the other clinical assessments like rotarod, visual acuity, and survival did not show clear differences among sex. This observation also argues for a variable treatment response of myeloid cells in different brain regions since our clinical readouts reflect neuropathological changes in various CNS compartments. Those readouts showing sex differences might thus be related to the function of CNS regions with distinct responses of males and females upon PLX3397 treatment. The observed bias in the susceptibility of different CNS regions toward microglia depletion in *Ppt1*^−/−^ mice might be reflected in the various reports of microglial subpopulations between brain regions which show different functional responses not only in development and health but also in disease [21, 37, 44, 47, 51, 53]. This diversity of microglial functionality is most likely dictated by a combination of ontogeny and local microenvironment and microglial activity is regionally dependent on multiple cues [2, 4, 12].

On the other hand, our data indicates that monocyte-derived myeloid cells seem to infiltrate the cortex but not retinotectal system of *Ppt1*^−/−^ mice, at least during the later stages of the disease. A simple explanation regarding the differential susceptibility of the cortical region to depletion could therefore be an increased infiltration and resistance of infiltrated cells to CSF-1R inhibition in the cortex. However, this hypothesis can neither explain the observed sex bias nor fully account for the observed low responsiveness of CD11b^+^ cells toward depletion in the cortical region. The above-mentioned regional sex bias is therefore more likely indicative of a sexual dimorphism in microglia. A similar sex-biased depletion efficacy upon PLX3397 treatment in the murine cortex has recently been reported to be independent of pharmacokinetic concentrations of the drug in the brain [15]. This is in line with various studies reporting remarkable sex differences in microglia regarding key cellular functions as well as transcriptome and proteome profiles in health and disease [14, 29, 31, 45, 55, 64, 69–71]. These findings are getting more attention recently because of the well-documented sex-related susceptibility to psychiatric and neurological diseases, in which neuroinflammation is a common denominator [39, 40, 59]. Such observations make it conceivable that differences in the transcriptome and proteome could result in a differential responsiveness of male and female microglia toward CSF-1R inhibition using PLX3397.

### High expression levels of homeostatic microglia markers correlate with an efficient depletion in *Ppt1*^−/−^ mice

During our investigations, we detected decreased expression levels of the CSF-1 receptor on CD11b^+^ cells in *Ppt1*^−/−^ mice compared to *Ppt1*^+/+^ controls as assessed by immunohistochemistry. This is in line with recent data showing that microglial signatures including specific transcriptional profiles and surface protein expression patterns in health and disease are highly plastic [7]. Among these differentially regulated proteins is the CSF-1R whose expression gets suppressed with microglial activation in disease [7, 37, 42]. Of note, also the downregulation of TMEM119 in the context of disease corresponds with our findings displaying a decrease in TMEM119 expression on a subset of CD11b^+^ cells in the CNS of *Ppt1*^−/−^ mice. Our study showed that ramified cells, found to have higher CSF-1R and TMEM119 expression levels, are more responsive to CSF-1R inhibition compared to amoeboid cells that showed very low CSF-1R expression levels. Also, hyper-ramified “bushy” microglia—possibly transitioning into the amoeboid form—showed a reduction of immunohistochemical CSF-1R and TMEM119 labeling. As these findings could only be partially explained by an infiltration of monocyte-derived myeloid cell, our observations imply that microglia downregulate their CSF-1R expression in *Ppt1*^−/−^ mice when transforming into activated states. Since the CSF-1 receptor is required for survival of microglia, a compensatory upregulation of other receptors allowing survival upon microglial activation is likely. TREM2 was shown to promote microglial survival by activating the Wnt/ß-catenin signaling pathway [74] and is known to be upregulated in disease-associated microglia [7, 37], possibly enabling them to “sense” signals related to neural damage [13]. The CSF-2/GM-CSF (granulocyte macrophage colony-stimulating factor) receptor complex, among others expressed on myeloid cells [33], and the upregulation of its ligand CSF-2 have been implicated in various neurological diseases like Alzheimer’s disease, multiple sclerosis, and vascular dementia [54, 67] and could possibly promote microglial survival as well. Interestingly, recent studies demonstrated that balanced CSF-1/CSF-2 receptor signaling is required for microglial homeostasis and that downregulation of CSF-1R expression results in a compensatory increase of CSF-2 in the CNS [10]. These combined observations are also supported by a study by Elmore et al. showing that microglia in aged wildtype mice are less sensitive toward depletion by CSF-1R inhibition suggesting an increased activation of compensatory pathways [16]. It is furthermore conceivable that these compensatory mechanisms are differentially pronounced among sex and CNS compartment providing another possible explanation for the sex- and compartment-related differences seen in CLN1 mice upon treatment. Indeed, based on recent findings, maintenance of microglia in white matter regions appears to be highly dependent on CSF-1, whereas interleukin-34 plays a more prominent role in maintenance of gray matter microglia [15]. Depletion of both CSF-1R ligands also resulted in a more efficient depletion of microglia in the white matter, again indicating alternative survival signals in the gray matter. Further studies are necessary to clarify the identity of these signals.

### Possible therapeutic considerations regarding CSF1-R inhibition

Similar to previous observations in models of hereditary CNS diseases [24, 35], pharmacological depletion of microglia attenuated disease outcome in CLN1 mice. This appears to contrast with other models of a demyelinating lysosomal storage disease [18] or Alzheimer’s disease [68], demonstrating that microglial reactions can be both beneficial and detrimental in distinct disease models or even at distinct disease stages [26, 65] and might reflect different activation states [61].

Albeit inhibition of CSF-1R on microglia is a valuable tool to study the role of microglia during pathogenesis and might serve as a therapeutic approach, it was reported that the CSF-1R is also expressed in selected neuron subpopulations providing important neuroprotective and survival signals in the neurodegenerative setting [49]. It was furthermore shown that CSF-1R haploinsufficiency is linked to adult-onset leukoencephalopathy with axonal spheroids and pigmented glia [11]. In addition, high treatment dosages and acute or extended durations of microglia depletion might interfere with the physiological functions of microglia in the brain and thereby result in detrimental effects [38, 46, 63].

These considerations are especially important for prospective translational approaches. Based on our present and previous observations [25, 27], it is likely that the beneficial effects of microglia depletion in CLN1 mice result from attenuation of neuroinflammatory reactions and especially CD8^+^ T cell-mediated damage. How exactly microglial activation and T cell-mediated neural damage are linked in CLN1 mice remains to be clarified. Nevertheless, since clinically well-established immunomodulators are available to target such adaptive immune reactions [22], the health risks associated with depleting microglia as a physiologically important cell population of the CNS must be carefully assessed; especially as they may depend on a plethora of conditions, like disease type, age, disease progression, intended brain region to target, and possibly sex. Alternative strategies to modulate the innate immune system and thereby indirectly suppress detrimental adaptive immune reactions might offer more feasible therapeutic perspectives [60]. Despite these considerations, the present study shows that targeting microglia by inhibition of the CSF-1R might offer one possible approach to attenuate deterioration of vision and improve quality of life in CLN1 disease.

## Conclusions

The present study demonstrates a detrimental contribution of innate immune reactions within disease course and outcome in a mouse model for CLN1 disease. We show that pharmacological depletion of microglia/macrophages in the CNS using the CSF-1R inhibitor PLX3397 significantly attenuates neuroinflammation and thereby ameliorates neurodegeneration and clinical outcome in *Ppt1*^−/−^ mice. Moreover, we describe a sex- and CNS compartment-related bias in depletion efficacy that correlates with the treatment outcome. These results provide insights into CLN pathogenesis and may guide in the design of immunomodulatory treatment strategies for the fatal lysosomal storage diseases.

## Supplementary information

**Additional file 1: Figure S1**. PLX3397 treatment inhibits pro-inflammatory cytokine expression in the CNS of CLN1 mice. **Figure S2**. Microglia/macrophages in distinct CNS compartments of CLN1 mice display morphological heterogeneity. **Figure S3**. Decreased expression of TMEM119 on CD11b+ cells in the cortex of CLN1 mice. **Figure S4**. Increased numbers of non-microglial myeloid cells in brains of CLN1 mice.

## Data Availability

All data generated during this study are included in this published article [and its supplementary information files]. Further details regarding the presented datasets are available from the corresponding author upon request.

## Abbreviations

APP: Amyloid precursor protein
CLN: Neuronal ceroid lipofuscinosis
PPT1: Palmitoyl-protein thioesterase 1
CSF-1R: Colony-stimulating factor-1 receptor
CSF-2: Colony-stimulating factor-2
OCT: Optical coherence tomography
PLP1: Proteolipid protein 1
S1Bf: Somatosensory barrel field cortex
Sn: Sialoadhesin
TMEM119: Transmembrane protein 119
Wt: Wildtype
