# Supplementary material for: Sex- and region-biased depletion of microglia/macrophages attenuates CLN1 disease in mice
Source: J Neuroinflammation. 2020 Oct 28;17:323. doi: 10.1186/s12974-020-01996-x (PMC7594417; doi:10.1186/s12974-020-01996-x)
Supplement: Supplementary file 1 — Additional file 1: Figure S1. PLX3397 treatment inhibits pro-inflammatory cytokine expression in the CNS of CLN1 mice. Figure S2. Microglia/macrophages in distinct CNS compartments of CLN1 mice display morphological heterogeneity. Figure S3. Decreased expression of TMEM119 on CD11b+ cells in the cortex of CLN1 mice. Figure S4. Increased numbers of non-microglial myeloid cells in brains of CLN1 mice. [file 12974_2020_1996_MOESM1_ESM.pdf]

Supplementary material

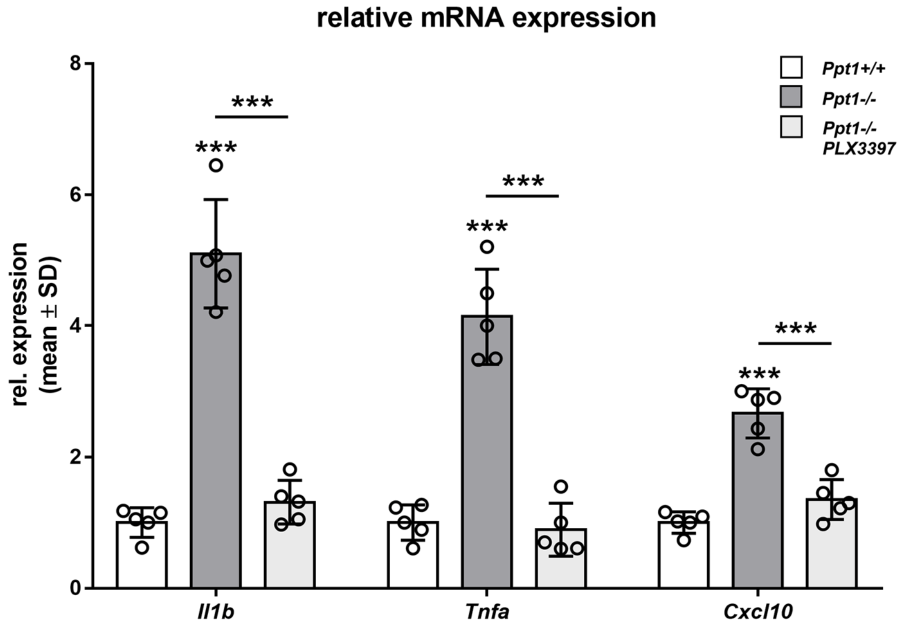

Supplementary Figure 1

PLX3397 treatment inhibits pro-inflammatory cytokine expression in the CNS of CLN1 mice.

Semiquantitative real-time PCR for relative *Il1b*, *Tnfa* and *Cxcl10* mRNA expression levels in optic nerves of 6-month-old male *Ppt1*$^{+/+}$, *Ppt1*$^{-/-}$, and PLX3397 treated *Ppt1*$^{-/-}$ mice. *Gapdh* expression was used as endogenous control and expression levels were related to the mean value of *Ppt1*$^{+/+}$ mice. PLX3397 inhibited the increased expression of *Il1b*, *Tnfa* and *Cxcl10* in optic nerves of *Ppt1*$^{-/-}$ mice (n = 5 mice per group). One-way ANOVA and Tukey's post hoc tests. ***$P < 0.001$.

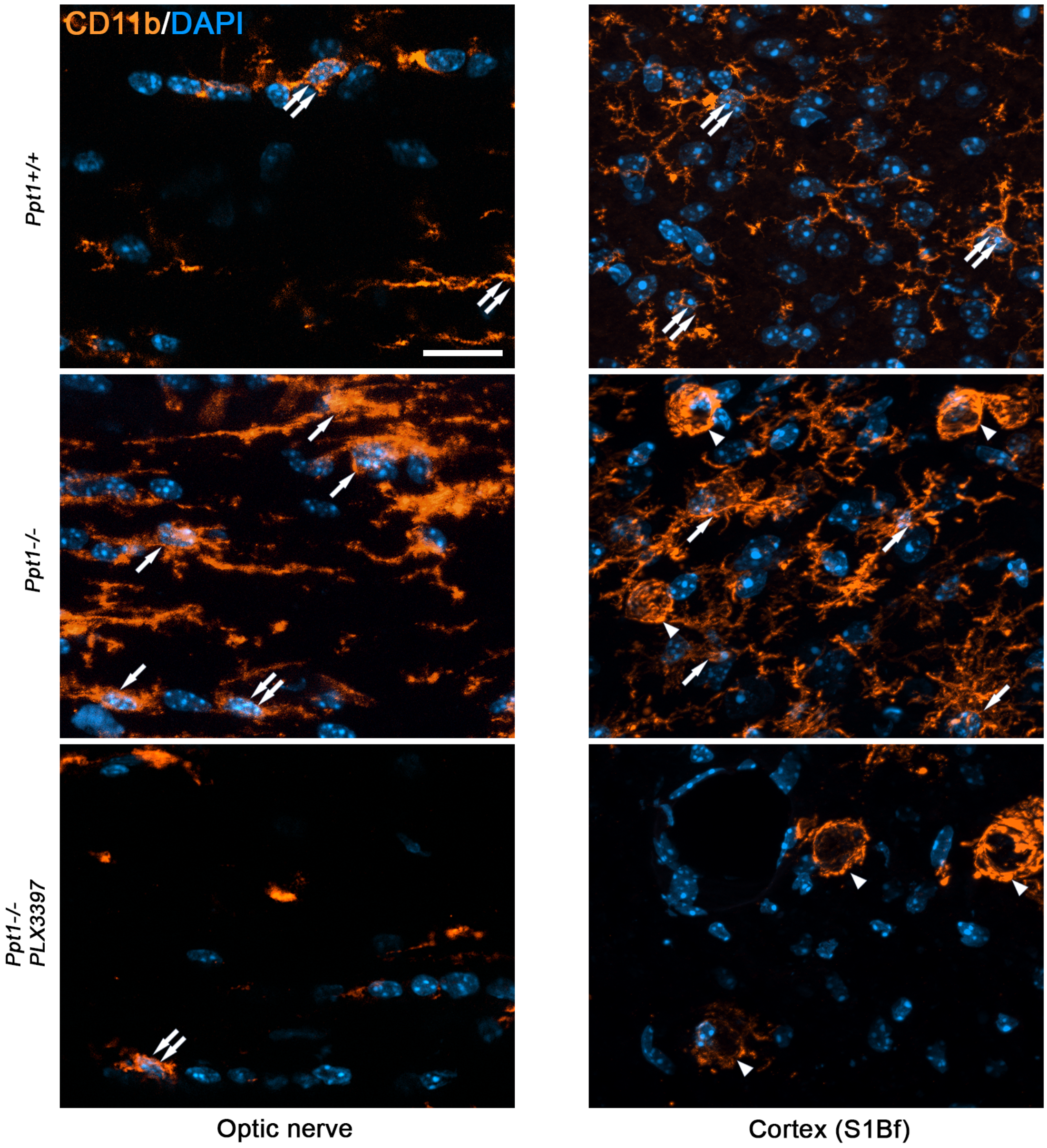

Supplementary Figure 2

Microglia/macrophages in distinct CNS compartments of CLN1 mice display morphological heterogeneity.

Immunofluorescent visualization of CD11b+ microglia/macrophages in longitudinal optic nerve (left) and S1Bf cortex sections (right) of 6-month-old *Ppt1*$^{+/+}$, *Ppt1*$^{-/-}$ mice and PLX3397 treated *Ppt1*$^{-/-}$ mice after 5 months of treatment. In *Ppt1*$^{+/+}$ mice double arrows indicate the typical homeostatic state of microglia with thin ramified processes, while in *Ppt1*$^{-/-}$ mice arrows depict either microglia with thicker "bushy" processes (optic nerve) or a hyper-ramified "bushy" phenotype (cortex). Arrowheads point towards round amoeboid microglia/macrophages. Note the absence of amoeboid cells in optic nerves of *Ppt1*$^{-/-}$ mice. Scale bar: 20 µm.

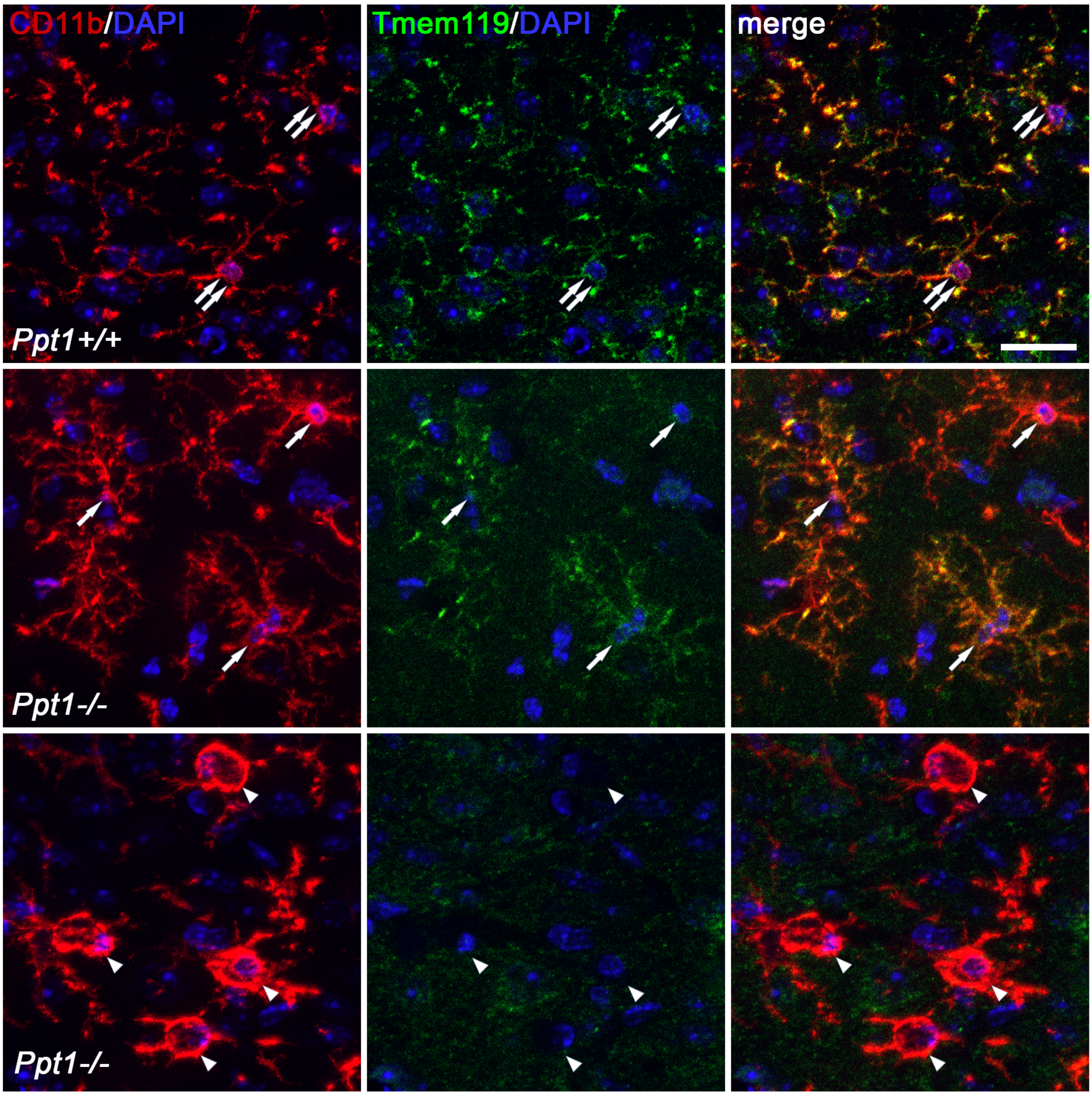

Supplementary Figure 3

Decreased expression of TMEM119 on CD11b+ cells in the cortex of CLN1 mice.

Representative fluorescent microscopic images of TMEM119 immunoreactivity on CD11b+ cells in the S1Bf cortex of *Ppt1*$^{+/+}$ (top) and *Ppt1*$^{-/-}$ (middle and bottom) mice. Virtually all CD11b+ microglia in *Ppt1*$^{+/+}$ mice were TMEM119+ (double arrows). Only amoeboid microglia/macrophages in *Ppt1*$^{-/-}$ *mice* were TMEM119- (arrowheads), while ramified "bushy" cells showed TMEM119 immunoreactivity (arrows). Scale bar: 20 µm.

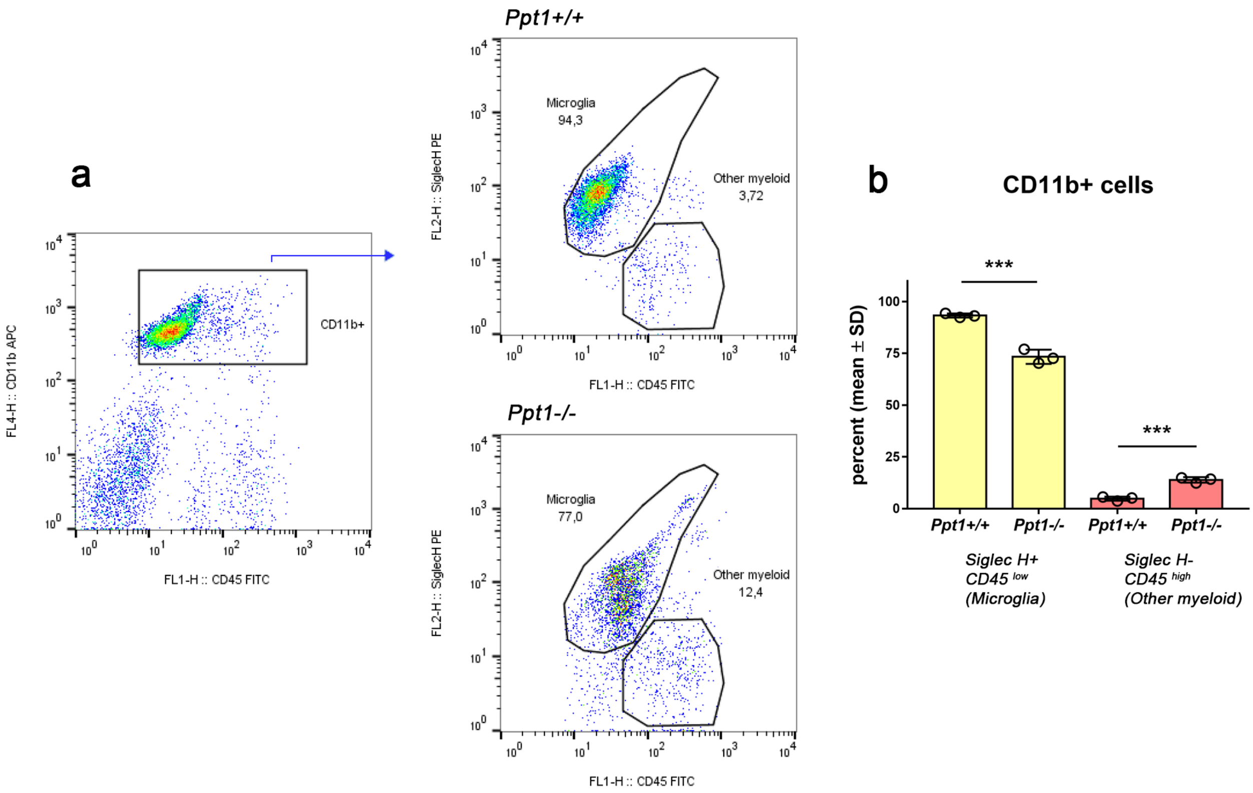

Supplementary Figure 4

Increased numbers of non-microglial myeloid cells in brains of CLN1 mice.

a, Representative flow cytometric analysis showing the gating strategy for single viable cells from whole brains of 6-month-old *Ppt1*$^{+/+}$ and *Ppt1*$^{-/-}$ mice: myeloid cells were gated based on their CD11b and CD45 expression followed by discrimination of Siglec H+ CD45low microglia and other Siglec H- CD45high myeloid cells. Note increased autofluorescence and CD45 expression by microglia from *Ppt1*$^{-/-}$ mice. **b,** Quantification of microglia and other myeloid cells in *Ppt1*$^{+/+}$ and *Ppt1*$^{-/-}$ mice, respectively. Two-tailed unpaired Student's *t*-test. $*P < 0.05$, $**P < 0.01$.
